# Orthogonal quasi-phase-matched superlattice for generation of hyperentangled photons

**DOI:** 10.1038/s41598-017-03023-1

**Published:** 2017-06-23

**Authors:** Salem F. Hegazy, Salah S. A. Obayya, Bahaa E. A. Saleh

**Affiliations:** 1 National Institute of Laser Enhanced Sciences, Cairo University, 12613 Giza, Egypt; 2 CREOL, The College of Optics & Photonics, University of Central Florida, Orlando, FL 32816 USA; 3 Center for Photonics and Smart Materials, Zewail City of Science and Technology, 12588 Giza, Egypt

## Abstract

A crystal superlattice structure featuring nonlinear layers with alternating orthogonal optic axes interleaved with orthogonal poling directions, is shown to generate high-quality hyperentangled photon pairs via orthogonal quasi-phase-matched spontaneous parametric downconversion. We demonstrate that orthogonal quasi-phase matching (QPM) processes in a single nonlinear domain structure correct phase and group-velocity mismatches concurrently. Compared with the conventional two-orthogonal-crystals source and the double-nonlinearity single-crystal source, the orthogonal QPM superlattice is shown to suppress the spatial and temporal distinguishability of the generated photon pairs by several orders of magnitude, depending on the number of layers. This enhanced all-over-the-cone indistinguishability enables the generation of higher fluxes of photon-pairs by means of the combined use of (a) long nonlinear crystal in noncollinear geometry, (b) low coherence-time pumping and ultra-wide-band spectral detection, and (c) focused pumping and over-the-cone detection. While each of these three features is challenging by itself, it is remarkable that the orthogonal QPM superlattice meets all of these challenges without the need for separate spatial or temporal compensation.

## Introduction

Hyperentanglement, the simultaneous entanglement in multiple degrees of freedom of a quantum system, has been widely used to circumvent limitations of linear optics^1^ in realizing various quantum operations like Bell-state analysis^2–7^, beating the channel capacity limit^8^, superdense teleportation^9^, deterministic entanglement purification^10, 11^, and teleportation of multiple degrees of freedom^12^. While higher-order entanglement can be implemented via either multipartite^13^ or multidimensional^14–18^ quantum systems, the latter is always much simpler to implement, manipulate, and measure^19, 20^. Hyperentangled photons, interpreted as high-dimensional quantum systems, enable faster and more-robust quantum computation using hyper-parallel quantum logic^21^.

A perfect source of hyperentangled photon pairs (biphotons) allows multiple possibilities for biphoton emissions that are indistinguishable in all degrees of freedom — spatial, spectral, and polarization — over its entire emission cone. If the source employs an anisotropic and dispersive medium of finite volume within which the emitted photons propagate and experience different spatial and spectral effects, then the emitted photons acquire features that label their spatial or temporal origin, thereby making perfect indistinguishability unlikely, if not impossible.

The simplest source of hyperentangled photon pairs is based on spontaneous parametric downconversion (SPDC) in a single type-II nonlinear crystal^22^. Entanglement is enabled by the multiple possibilities of satisfying energy and momentum conservation for the orthogonal polarization. The type-II process exhibits polarization and spectral entanglement within small solid angles centered about two specific noncollinear directions defined by the intersection of two emission cones with offset axes. The bandwidth of spectral entanglement is limited by the phase matching condition, which can often be aided by use of periodically poled crystals that introduce quasi-phase matching (QPM).

A more efficient source of hyperentangled biphotons uses two abutted thin nonlinear crystals with orthogonal optic axes, each generating type-I SPDC with the same pump^8, 9, 18, 23–35^. When the pump is polarized at 45° the SPDC emissions from the two crystals have orthogonal polarization. Since the crystals are thin, it is not possible to determine from which crystal an emitted photon pair originates, so that indistinguishability extends over an entire ring with axis along the pump beam, instead of the intersection of two offset rings. A principal limitation of this *cascaded-crystals* (CC) source is that the nonlinear crystals must be rather thin.

Certain physical effects limit or destroy the spatial and temporal indistinguishability of the emitted photons in these sources. Spatial distinguishability originates primarily from the transverse walkoff and the noncollinear geometry^23^. Temporal distinguishability is caused by material birefringence and dispersion, which lead to group-velocity mismatch between the orthogonal polarizations accompanied by longitudinal walkoff. Spatial distinguishability can be reduced by use of a pump beam of waist much wider than the thickness of the crystal(s), but this comes at the expense of diminishing the strength of the nonlinear interaction. However, a focused pump does not create SPDC cones with full indistinguishability, so that high-quality entanglement is constrained to narrow collection angles. Temporal indistinguishability may be restored by making use of compensation, such as additional birefringent element(s) that compensate for the longitudinal walkoff acquired inside the nonlinear material. This type of compensation is always necessary, particularly for long crystal(s) or when the pump has a low coherence time^25–28, 30–33^. Nevertheless, compensation for spatial labeling becomes inadequate when entanglement is desired over wide spatial and spectral windows^24, 32–34^.

Another source of hyperentangled biphotons uses a single crystal endowed with a nonlinear tensor supporting two nonlinear type-I and type-0 processes concurrently, so that when pumped by a linearly polarized beam, photon pairs with two orthogonal polarization are emitted from any location along the crystal^36^. Concurrent phase matching for these processes requires temperature tuning and periodic poling of the crystal that enable equal and overlapped noncollinear emissions from type-I and type-0 processes. This source is referred to hereafter as the *double-nonlinearity* (DN) source.

**Figure 1:**
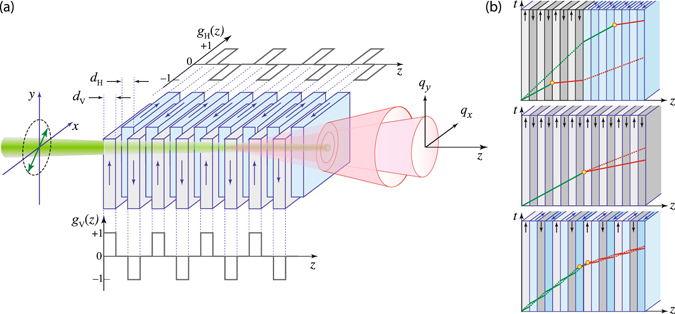
(**a)** Schematic of the superlattice structure showing the two interleaved sets of nonlinear layers with orthogonal optic axes and orthogonal directions of poling, as indicated by the *g*_*H*_(*z*) and *g*_*V*_(*z*) functions. The arrows show the periodically flipped directions of nonlinearity for each set along the crystal domains, which implement the orthogonal QPM processes. When illuminated by a linearly polarized pump beam with components along the horizontal and vertical directions, parametric downconversion creates in photon pairs with alternating orthogonal polarizations. (**b**) Space-time diagrams illustrating the propagation of biphotons born in cascaded-crystals (top), double-nonlinearity (middle), and superlattice (bottom) structures (dotted line: *o* ray; solid line: *e* ray). The orthogonal poling in the superlattice performs continuous group-velocity matching per two-layer set for the pump (green line) and downconverted (red line) photons.

In this paper, we present a new SPDC source of all-over-the-cone hyperentangled biphotons using a periodic thin-layered structure with alternating orthogonal optic axes, combined with periodic poling along orthogonal directions. This layered structure may be regarded as two identical periodically poled crystals with empty gaps separating adjacent layers: one crystal is rotated 90° and inserted into the empty gaps of the other, much like a mitre dovetail joint^37^, as illustrated in Fig. 1. By combining periodic downconversion of orthogonally polarized photons along with periodic poling that corrects the phase mismatch, the structure self corrects for longitudinal walkoff as it happens and before it accumulates. Each of the two orthogonal sets of nonlinear layers acts like spacers that engineer the group-delay^38, 39^ of the emission of the other set. QPM is woven into each of the orthogonally polarized emissions, and as in other SPDC processes it enables access to the highest nonlinear coefficient, opens the possibility for tunability with highly reduced bandwidth over a wide spectral range^40^, and offers a unique capability of tailoring the spatiotemporal properties of the entangled photons^41–45^. In comparison with the CC source and the DN source, this superlattice (SL) structure is shown to emit SPDC biphotons with nearly perfect spatial and temporal indistinguishability over ultra-wide spatial and spectral windows, even if it uses a focused and low-coherence-time beam to pump a long structure in a noncollinear interaction geometry, and without the need for compensating elements.

## Results

### Quantum state

The nonlinear domain structure proposed in this paper is made of *M* contiguous parallel layers constructed by interleaving two sets of layers with orthogonal optic axes pointing along the *x* and *y* axes, as illustrated in Fig. 1a, and referred heretofore as the H and V layers. Elements of the H and V layers have thicknesses *d*_H_ and *d*_V_, respectively, so that the overall length of the structure is $L=\frac{1}{2}({d}_{{\rm{H}}}+{d}_{{\rm{V}}})M$. We consider the parametric downconversion of an intense linearly polarized pump beam propagating along the axis of the structure (the *z* axis) in the type-0 (eee) regime. The interacting component of the pump field, the signal, and the idler fields are linearly polarized along the optic axes of each of the layers. The SPDC process is enhanced by means of QPM using periodic poling of the H and V layers along their respective optic axes.

This alternating structure may also be regarded as a unit set made of a pair of successive layers, forming a cascade of crossed layers, repeated *M*/2 times. The thickness of this two-layer unit set is *d* = *d*_H_ + *d*_V_, and the overall length is *L* = *Md*/2. Each two-layer unit set supports nonlinear interactions in both the vertical (V) and the horizontal (H) directions, in sequence. Embedded in this overall structure is a periodic reversal of the nonlinearity sign, which enables concurrent QPM for both the H and V polarizations, in a distributed coherent fashion.

The alternation of the H and V layers suppresses the temporal which-layer information by realizing group-velocity matching between the HH and VV biphotons, thereby limiting their longitudinal walkoffs, as illustrated in the space-time diagrams in Fig. 1b, which demonstrates the clear superiority of the superlattice structure. In the space domain, the suppression of which-layer information is realized when the emission angle is much smaller than the ratio of the pump waist to the domain thickness^23^. This is also applicable in the presence of transverse walkoff. For the superlattice structure, this ratio is extremely large (thickness is in the order of few micrometers) compared with the cascaded implementations, where the thickness is the crystal length *L* itself.

The pump beam is treated classically as a superposition of monochromatic plane waves and its H and V components are expressed as ${E}_{p}^{(H,V)}({\bf{x}},z,t)={\sigma }_{H,V}\int d{\omega }_{p}d{{\bf{q}}}_{p}\,{A}_{p}({\omega }_{p};{{\bf{q}}}_{p})\exp \,i({\kappa }_{p}z+{{\bf{q}}}_{p}.{\bf{x}}-{\omega }_{p}t)+{\rm{c}}{\rm{.c}}{\rm{.}}$, with angular frequencies *ω*_*p*_ and wave vectors **k**_*p*_ = (**q**_*p*_, *κ*_*p*_) where **q**_*p*_ are the components along the transverse coordinates **x** = (*x*, *y*), *κ*_*p*_ are the longitudinal components, and *σ*_*H*,*V*_ are the relative amplitudes of H and V polarization components.

The downconverted waves are also expressed in similar spectral and spatial expansions in terms of monochromatic planar waves with angular frequencies *ω*_1,2_ and wave vectors **k**_1,2_ = (**q**_1,2_, *κ*_1,2_), traveling through the sequence of uniaxial anisotropic layers of the domain. Maxwell’s equations dictate that ${\kappa }_{j}^{o}({\omega }_{j};{{\bf{q}}}_{j})=\sqrt{{({\omega }_{j}{n}_{j}^{o}/c)}^{2}-{|{{\bf{q}}}_{j}|}^{2}}$ for the ordinary (*o*) polarized field and ${\kappa }_{j}^{e(H,V)}({\omega }_{j};{{\bf{q}}}_{j})=\sqrt{{({\omega }_{j}{n}_{j}^{e}/c)}^{2}-{|{{\bf{B}}}_{j}^{H,V}{{\bf{q}}}_{j}|}^{2}},j=p,\,\mathrm{1,}\,2$, for the extraordinary (*e*) polarized field^46, 47^, within the H and V layers, where ${n}_{j}^{o}$ and ${n}_{j}^{e}$ are the principal values of the refractive index and $${{\bf{B}}}_{j}^{H}=[\begin{array}{cc}{n}_{j}^{e}/{n}_{j}^{o} & 0\\ 0 & 1\end{array}],\quad {{\bf{B}}}_{j}^{V}=[\begin{array}{cc}1 & 0\\ 0 & {n}_{j}^{e}/{n}_{j}^{o}\end{array}]$$. Along the alternating structure, the longitudinal wave numbers ${\kappa }_{j}^{e(H,V)}$ are generally different because of the astigmatic terms ${|{{\bf{B}}}_{j}^{H,V}{{\bf{q}}}_{j}|}^{2}$.

The horizontal and vertical components of the pump generate H and V downconverted photon pairs from the successive H and V layers of the structure, respectively, and the design of the domain structure is such that it is equally probable for a pump photon to downconvert in any of the *M* layers in one of the two polarizations. The superimposed SPDC radiation creates an entangled state described at small emission angles^48^ by the superposition

1 $$\begin{array}{rcl}|\psi \rangle  & = & \int d{\omega }_{1}d{\omega }_{2}d{{\bf{q}}}_{1}d{{\bf{q}}}_{2}\{{{\rm{\Phi }}}_{{\rm{H}}}({\omega }_{1},{\omega }_{2};{{\bf{q}}}_{1},{{\bf{q}}}_{2})|H;{\omega }_{1};{{\bf{q}}}_{1}\rangle |H;{\omega }_{2};{{\bf{q}}}_{2}\rangle \\  &  & +\,{{\rm{\Phi }}}_{{\rm{V}}}({\omega }_{1},{\omega }_{2};{{\bf{q}}}_{1},{{\bf{q}}}_{2})|V;{\omega }_{1};{{\bf{q}}}_{1}\rangle |V;{\omega }_{2};{{\bf{q}}}_{2}\rangle \},\end{array}$$

where Φ_H,V_(*ω*_1_, *ω*_2_; **q**_1_, **q**_2_) are normalized weight functions representing the two-photon amplitudes over all spatial and spectral modes. Each pump plane-wave component is responsible for the generation of a pair of downconverted waves, perfectly satisfying the conservation of energy (*ω*_1_ + *ω*_2_ = *ω*_*p*_) and transverse momentum (**q**_1_ + **q**_2_ = **q**_*p*_). The two-photon amplitudes can therefore be written as (see Methods)

2 $${{\rm{\Phi }}}_{{\rm{H}},{\rm{V}}}({\omega }_{1},{\omega }_{2};{{\bf{q}}}_{1},{{\bf{q}}}_{2})\propto {\sigma }_{{\rm{H}},{\rm{V}}}{d}_{{\rm{H}},{\rm{V}}}{\chi }^{\mathrm{(2)}}{A}_{p}({\omega }_{1}+{\omega }_{2};{{\bf{q}}}_{1}+{{\bf{q}}}_{2}){\rm{sinc}}(\frac{1}{2}{\rm{\Delta }}{\kappa }_{{\rm{H}},{\rm{V}}}^{e}{d}_{{\rm{H}},{\rm{V}}}){{\rm{S}}}_{M}(\frac{1}{2}{\rm{\Delta }}{\phi }_{{\rm{H}},{\rm{V}}}),$$

where *χ* ^(2)^ is the second-order susceptibility of the bulk domain material, ${\rm{\Delta }}{\kappa }_{H,V}^{e}={\kappa }_{p}^{e(H,V)}-{\kappa }_{1}^{e(H,V)}-{\kappa }_{2}^{e(H,V)}$ and ${\rm{\Delta }}{\kappa }^{o}={\kappa }_{p}^{o}-{\kappa }_{1}^{o}-{\kappa }_{2}^{o}$ are the errors in satisfying the longitudinal conservation of momentum within the interacting (H or V) layers and the noninteracting layers, respectively, ${\rm{sinc}}(x)\,\equiv \,\sin (x)/x$, and ${S}_{M}(x)\,\equiv \,\sin (\tfrac{1}{2}Mx)/\sin \,x$ is the phased-array function, which has a maximum value of *M*/2 for *x* = 0. This corresponds to satisfying the QPM conditions

3 $${\rm{\Delta }}{\phi }_{{\rm{H}},{\rm{V}}}={\rm{\Delta }}{\kappa }^{o}{d}_{{\rm{V}},{\rm{H}}}+{\rm{\Delta }}{\kappa }_{{\rm{H}},{\rm{V}}}^{e}{d}_{{\rm{H}},{\rm{V}}}-\mu \pi =\mathrm{0,}$$

for HH and VV emissions, respectively, where *μ* is an integer representing the order of QPM. The layered structure therefore tailors the spatiotemporal two-photon wavepacket by introducing the phased-array factors ${{\rm{S}}}_{M}(\frac{1}{2}{\rm{\Delta }}{\phi }_{{\rm{H}},{\rm{V}}})$, which are much narrower (for *M* ≫ 1) than the bulk phase-matching functions ${\rm{sinc}}({\rm{\Delta }}{\kappa }_{{\rm{H}},{\rm{V}}}^{e}{d}_{{\rm{H}},{\rm{V}}}\mathrm{/2)}$. The phase matching conditions in equation (3) determine the layers thicknesses *d*_H,V_ required to obtain superimposed H and V SPDC cones centered at some particular emission angle.

The polarization, spatial, and spectral variables in the biphoton quantum state described by equation (1) are inherently coupled so that its hyperentangled nature cannot be directly observed. Nevertheless, the entanglement of one degree of freedom can be assessed by tracing out the two other degrees of freedom, as we will subsequently show.

Ideally, for maximally entangled polarization, the complex superposition weights Φ_H_ and Φ_V_ should be equal for all frequencies and transverse wave vectors. In reality, they are not, and their relative phase

4 $$\vartheta =\arg \{{{\rm{\Phi }}}_{{\rm{V}}}/{{\rm{\Phi }}}_{{\rm{H}}}\}=-{\textstyle \tfrac{1}{2}}{\rm{\Delta }}{\kappa }^{o}d-{\textstyle \tfrac{M}{4}}({\rm{\Delta }}{\phi }_{{\rm{V}}}-{\rm{\Delta }}{\phi }_{{\rm{H}}})$$

plays a major role in limiting the indistinguishability of the two components of the state.

Equations similar to equation (2) apply to the CC source, which has two cascaded periodically-poled orthogonal crystals featuring type-0 downconversion, albeit with a different QPM condition

5 $${\rm{\Delta }}{\phi }_{{\rm{H}},{\rm{V}}}^{(\mathrm{CC})}={\rm{\Delta }}{\kappa }_{{\rm{H}},{\rm{V}}}^{e}{d}_{{\rm{H}},{\rm{V}}}-\mu \pi =\mathrm{0,}$$

and relative phase

6 $${\vartheta }^{(\mathrm{CC})}=-\frac{1}{2}{\rm{\Delta }}{\kappa }^{o}L-\frac{M}{4}({\rm{\Delta }}{\phi }_{{\rm{V}}}^{(\mathrm{CC})}-{\rm{\Delta }}{\phi }_{{\rm{H}}}^{(\mathrm{CC})})\mathrm{.}$$

Note that the first term in equation (6) is much greater than the second (phase-matching) term, and generally dominates within the significant spectral and spatial regions.

For the DN source^36^, the concurrent type-0 and type-I interactions in a single periodically poled crystal produce the superposed HH and VV possibilities, respectively. The magnitude of the VV amplitude is therefore similar to the cascaded-crystals case [including the QPM condition ${\rm{\Delta }}{\phi }_{V}^{(\mathrm{DN})}$], and the HH amplitude is given by

7 $${{\rm{\Phi }}}_{{\rm{H}}}^{(\mathrm{DN})}({\omega }_{1},{\omega }_{2};{{\bf{q}}}_{1},{{\bf{q}}}_{2})\propto {\sigma }_{{\rm{V}}}{\chi }^{\mathrm{(2)}}{A}_{p}({\omega }_{1}+{\omega }_{2};{{\bf{q}}}_{1}+{{\bf{q}}}_{2})L\,{\rm{sinc}}(\frac{1}{2}{\rm{\Delta }}{\kappa }^{ooe}L),$$

with the relative phase be

8 $${\vartheta }^{(\mathrm{DN})}=\frac{1}{2}{\rm{\Delta }}{\kappa }^{ooe}L-\frac{M}{2}{\rm{\Delta }}{\phi }_{{\rm{V}}}^{(\mathrm{DN})},$$

where Δ*κ* ^*ooe*^ is the longitudinal wave vector mismatch for type-I (*ooe*) interaction, which is canceled out in the central emission direction via temperature tuning.

We now compare the polarization and spatiotemporal characteristics of the hyperentangled photons emitted by the three sources. For comparison on an equal footing, all structures are made of LiNbO_3_ crystals of 5-mm length featuring third-order QPM. We consider a typical experiment with a pump centered at 532 nm illuminating each of the three structures and producing nondegenerate and noncollinear SPDC light centered at 810 nm (signal) and 1550 nm﻿ (idler). This example is of practical importance because the range of the signal photon is appropriate for either quantum memory via atomic ensemble interaction^49, 50^ or low-noise highly efficient detection using commercial silicon avalanche photodiode, while the idler photon features low-loss long-distance fiber communication within the third telecom window^51, 52^. The QPM conditions in equations (3) and (5) give the design thicknesses *d*_H_ ≈ *d*_V_ with values 10.8 μm for the CC and DN sources, and 5 μm for the superlattice, all at a phase-matching temperature *T* = 114.41 °C. The pump is assumed to be pulsed with the normalized amplitude^53^

9 $${A}_{p}({\omega }_{p};{{\bf{q}}}_{p})=(\sqrt{{\tau }_{p}}/\sqrt[4]{\pi })\exp [-\frac{1}{2}{\tau }_{p}^{2}{({\omega }_{p}-{\omega }_{p}^{0})}^{2}]({W}_{p}/\sqrt{\pi })\exp (-\frac{1}{2}{W}_{p}^{2}{|{{\bf{q}}}_{p}|}^{2}),$$

which has separable spectral and spatial dependence. Here, ${\omega }_{p}^{0}$ is the central frequency, *τ*_*p*_ is the pulse duration, and *W*_*p*_ is the pump beam waist. For a planar pump we take *W*_*p*_ to be very large.

### Polarization characteristics

**Figure 2:**
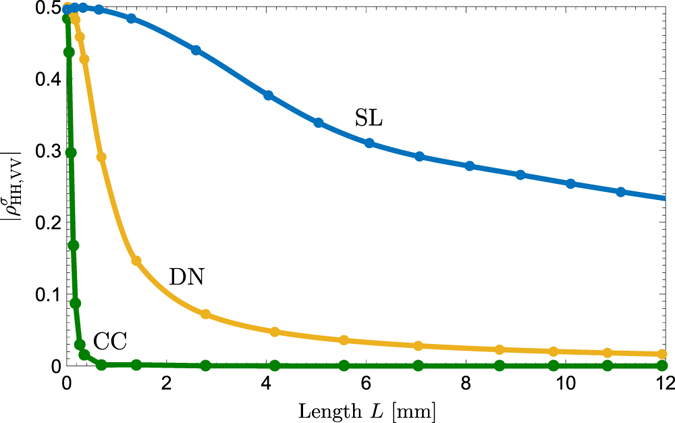
Comparison of the magnitude of the off-diagonal element ${\rho }_{HH,VV}^{\sigma }$ of the reduced density-matrix for biphotons emitted over the entire cone by three LiNbO_3_ structures—cascaded crystals (CC), double-nonlinearity (DN), and superlattice (SL)—excited by a 532-nm, 133-fs pulsed pump focused to a 50-μ﻿m ﻿waist. The downconversion is nondegenerate with signal and idler photons centered at 810 nm and 1550 nm and noncollinear with central emissions at the respective angles 0.5° and 0.96° in the free space. No spectral or spatial filters are used.

The quantum state (1) is a pure state in multiple degrees of freedom (polarization, spatial, and spectral) described by a density operator *ρ* with Tr(*ρ* ^2^) = 1. If the spatial and spectral degrees of freedom are traced out, the result is a mixed polarization state described by a reduced density operator *ρ* ^*σ*^ where Tr[(*ρ* ^*σ*^)^2^] < 1, only if correlations exist between polarization and the removed degrees of freedom^54, 55^ (see Methods). Since ${\rm{T}}{\rm{r}}[{({\rho }^{\sigma })}^{2}]=\frac{1}{2}+\frac{1}{2}{\vee }^{2}$, where $\vee =2|{\rho }_{\mathrm{HH},\mathrm{VV}}^{\sigma }|$ is the visibility of polarization entanglement^26^, the off-diagonal element of the reduced polarization density matrix ${\rho }_{\mathrm{HH},\mathrm{VV}}^{\sigma }$ is a measure of the partial purity or the indistinguishability of the HH and VV photon pairs. This element is given by

10 $${\rho }_{\mathrm{HH},\mathrm{VV}}^{\sigma }={({\rho }_{\mathrm{VV},\mathrm{HH}}^{\sigma })}^{\ast }=\int d{\omega }_{1}d{\omega }_{2}d{{\bf{q}}}_{1}d{{\bf{q}}}_{2}{{\rm{\Phi }}}_{{\rm{H}}}({\omega }_{1},{\omega }_{2};{{\bf{q}}}_{1},{{\bf{q}}}_{2}){{\rm{\Phi }}}_{{\rm{V}}}^{\ast }({\omega }_{1},{\omega }_{2};{{\bf{q}}}_{1},{{\bf{q}}}_{2}),$$

so that it represents the degree of spatial and spectral overlap of Φ_H_ and Φ_V_. This purity measure is plotted in Fig. 2 as a function of the structure length *L* for each of the three sources. The integrals in equation (10) cover the entire SPDC emission with no spatial or spectral windows used. At its maximum of $\frac{1}{2}$, the off-diagonal element describes a pure (completely indistinguishable) polarization state, while at zero it corresponds to a mixed and entirely distinguishable polarization state. It is evident that the superlattice is far superior to both the CC and DN sources since it relatively maintains indistinguishability over longer structures, thereby enabling the generation of much higher biphoton fluxes with greater purity.

### Angular characteristics

**Figure 3:**
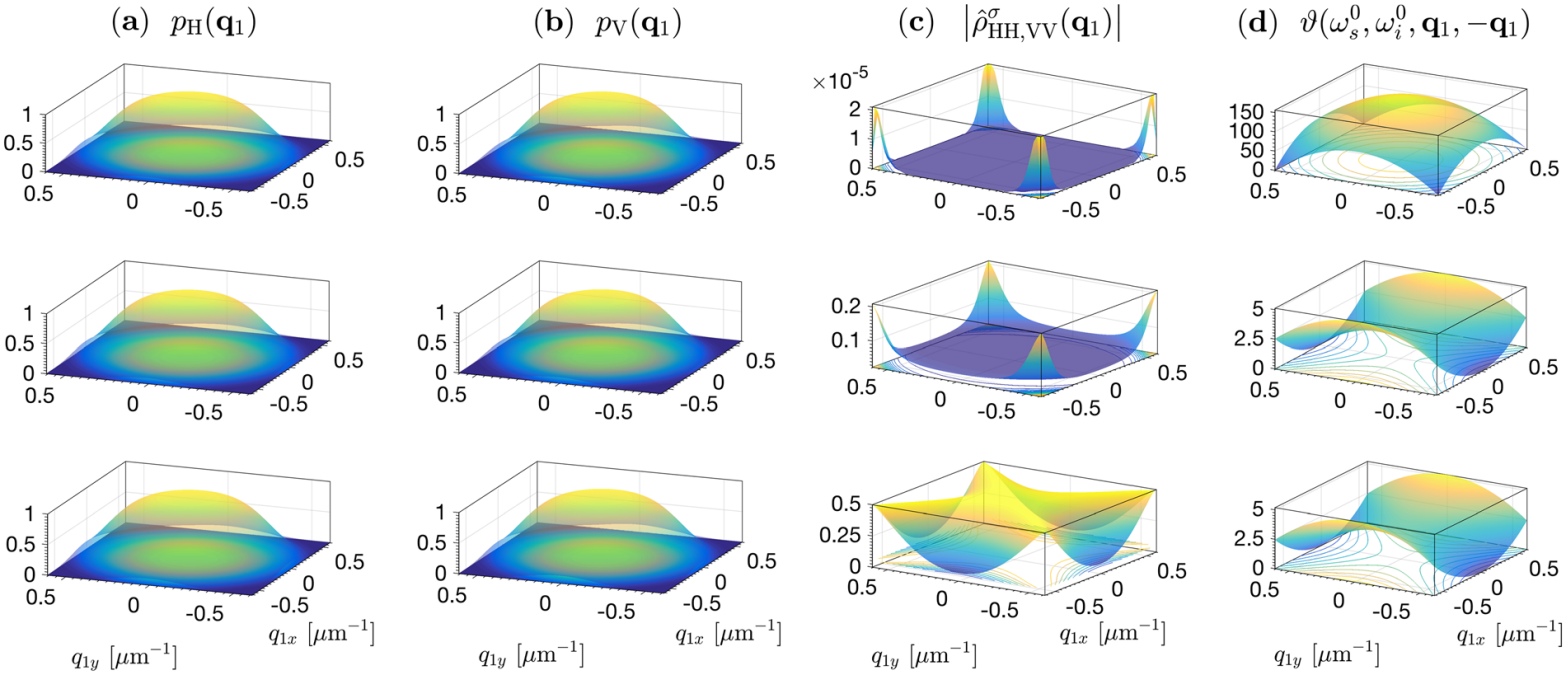
Comparison of the angular characteristics of biphotons emitted by three LiNbO_3_ structures—cascaded crystals (top), double-nonlinearity (middle), and superlattice (bottom)— of 5-mm overall length, excited by a plane-wave 133-fs pulsed pump at 532 nm. The downconversion is nondegenerate with signal and idler photons centered at 810 nm and 1550 nm, and noncollinear with central emission angles 0.5° and 0.96° in free space, respectively. Ultra-wide band spectral filters (70-nm full width at half maximum) are used. (**a**) Angular distribution of the signal emission cones, described by the function *p*_H_(**q**_1_) which equals the probability density *P*_H_(**q**_1_, **q**_2_) evaluated at **q**_2_ = −**q**_1_. (**b**) Same as in (**a**) for the VV component. (**c**) Magnitude of the cross element of the reduced density matrix ${\hat{\rho }}_{\mathrm{HH},\mathrm{VV}}^{\sigma }({{\bf{q}}}_{1})$. (**d**) Relative phase *ϑ* [rad] between the superposition weights of the |*HH*〉 and |*VV*〉 states, evaluated at **q**_2_ = −**q**_1_ as a function of **q**_1_ = (*q*_1*x*_, *q*_1*y*_). An overall phase offset is subtracted for clarity.

Equation (1) describes the entire SPDC field emerging from the nonlinear structure into free space. To delineate the angular (directional) characteristics, we use two spectral filters with Gaussian transmission $G(\omega )=(\mathrm{1/}\sqrt[4]{\pi {\sigma }_{\omega }^{2}})\exp (-{\omega }^{2}\mathrm{/2}{\sigma }_{\omega }^{2})$ and spectral width *σ*_*ω*_ centered at the signal and idler central frequencies ${\omega }_{\mathrm{1,2}}^{0}$ and placed directly after the nonlinear structure. The polarization-analyzed outcomes have the angular distributions

11 $${P}_{{\rm{H}},{\rm{V}}}({{\bf{q}}}_{1},{{\bf{q}}}_{2})=\int d{\omega }_{1}d{\omega }_{2}{|{{\rm{\Phi }}}_{{\rm{H}},{\rm{V}}}({\omega }_{1},{\omega }_{2};{{\bf{q}}}_{1},{{\bf{q}}}_{2})G({\omega }_{1}-{\omega }_{1}^{0})G({\omega }_{2}-{\omega }_{2}^{0})|}^{2},$$

which represent the probability densities of photon coincidence at points in the far field corresponding to the transverse wave vectors **q**_1_ and **q**_2_. Under a planar pump assumption **q**_2_ ≈ −**q**_1_, so that *P*_H,V_(**q**_1_, **q**_2_) are completely defined by the functions *p*_H,V_(**q**_1_) = *P*_H,V_(**q**_1_, −**q**_1_), which are plotted in Fig. 3a,b in the *xz* plane for the three structures.

The angular distribution of the relative phase *ϑ* = arg{Φ_V_/Φ_H_} is plotted in Fig. 3d. Ideally, this surface is flat. The plot shows that both the superlattice and the double-nonlinearity crystals have relative-phase angular distributions that are much flatter than that of the cascaded crystals.

Also shown in Fig. 3c is the distribution of the off-diagonal element of the reduced density matrix,

12 $${R}_{\mathrm{HH},\mathrm{VV}}^{\sigma }({{\bf{q}}}_{1},{{\bf{q}}}_{2})=\frac{\int d{\omega }_{1}d{\omega }_{2}{{\rm{\Phi }}}_{{\rm{H}}}({\omega }_{1},{\omega }_{2};{{\bf{q}}}_{1},{{\bf{q}}}_{2}){{\rm{\Phi }}}_{{\rm{V}}}^{\ast }({\omega }_{1},{\omega }_{2};{{\bf{q}}}_{1},{{\bf{q}}}_{2})}{2{[\int d{\omega }_{1}d{\omega }_{2}{|{{\rm{\Phi }}}_{{\rm{H}}}({\omega }_{1},{\omega }_{2};{{\bf{q}}}_{1},{{\bf{q}}}_{2})|}^{2}\int d{\omega }_{1}d{\omega }_{2}{|{{\rm{\Phi }}}_{{\rm{V}}}({\omega }_{1},{\omega }_{2};{{\bf{q}}}_{1},{{\bf{q}}}_{2})|}^{2}]}^{\frac{1}{2}}},$$

where ${\hat{\rho }}_{\mathrm{HH},\mathrm{VV}}^{\sigma }({{\bf{q}}}_{1})={R}_{\mathrm{HH},\mathrm{VV}}^{\sigma }({{\bf{q}}}_{1},-{{\bf{q}}}_{1})$. For the cascaded and double-nonlinearity crystals, this parameter is tiny (averages are 4.4 × 10^−7^ and 4.9 × 10^−2^, respectively), indicating a strong correlation between polarization and other degrees of freedom, which are particularly manifested under the challenging conditions considered here (long crystal, noncollinear geometry, wide spectral filter, and low-coherence-time pump). The higher values of $|{\hat{\rho }}_{{\rm{H}}{\rm{H}},{\rm{V}}{\rm{V}}}^{\sigma }({{\bf{q}}}_{1})|$ near the edges of the SPDC cone may be attributed to the relatively weaker birefringence, but these are not relevant since the rates of emission are negligible in this region.

In contrast, the superlattice exhibits $|{\hat{\rho }}_{{\rm{HH}},{\rm{VV}}}^{\sigma }({{\bf{q}}}_{1})|$ that is very close to its maximum value of 0.5 over a wide range, yielding an average about 0.4 over the entire emission cone. This remarkably high purity, particularly, near the central and diagonal regions, is attributed to the temporal indistinguishability resulting from group velocity matching, and the spatial indistinguishability, which is due to the approximately symmetric condition ${\kappa }_{j}^{e(H)}\approx {\kappa }_{j}^{e(V)}$ implicit in the dispersion relations for **q**_*j*,*x*_ ≈ **q**_*j*,*y*_ (*j* = 1, 2, *p*). Analogous symmetries can then emerge for other anisotropy-affected quantities like ${\rm{\Delta }}{\kappa }_{{\rm{H}},{\rm{V}}}^{e}$ and Δφ_H,V_, assuring the almost full symmetry of the two-photon wave functions Φ_H,V_(*ω*_1_, *ω*_2_; **q**_1_, **q**_2_). Realizing such a high value of $|{\hat{\rho }}_{{\rm{HH}},{\rm{VV}}}({{\bf{q}}}_{1})|$ reveals, as will be seen later, an underlying near-perfect temporal indistinguishability.

**Figure 4:**
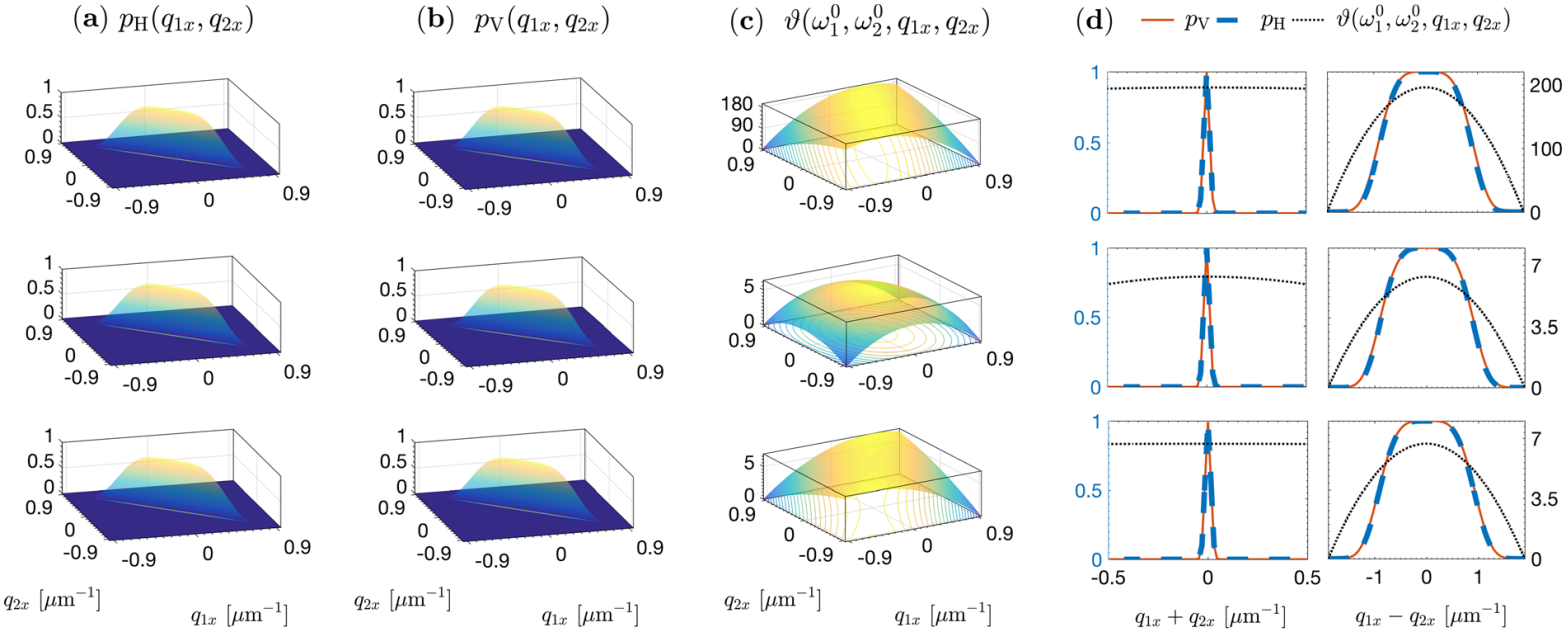
Comparison of the angular characteristics of biphotons emitted by three LiNbO_3_ structures—cascaded crystals (top), double-nonlinearity (middle), and superlattice (bottom)— of 5-mm overall length, excited by a 532-nm, 133-fs pulsed pump focused to a 50-μ﻿m waist. The downconversion is nondegenerate with signal and idler photons centered at 810 nm and 1550 nm, and noncollinear with central emission angles 0.5° and 0.96° in free space, respectively. Ultra-wide band spectral filters (70-nm full width at half maximum) are used. (**a**) Cross-section of the probability distribution *P*_H_(**q**_1_, **q**_2_) in the *x*–*z* plane: *p*_H_(*q*_1*x*_, *q*_2*x*_) = *P*_H_(*q*_1*x*_, 0, *q*_2*x*_, 0) for the HH component of the state. (**b**) Same as in (**a**) for the VV component. (**c**) Relative phase *ϑ* [rad] between the superposition weights of the |*HH*〉 and |*VV*〉 states, evaluated in the *x*–*z* plane as a function of *q*_1*x*_ and *q*_2*x*_. An overall phase offset is subtracted for clarity. (**d**) Normalized projections of the distributions in (**a**–**c**) along the diagonal and off-diagonal directions as functions of (*q*_1*x*_ + *q*_2*x*_) and (*q*_1*x*_ − *q*_2*x*_), normalized to their respective peak values.

The angular characteristics are compared in Fig. 4 for a focused pump. The angular distributions of the coincidence probabilities, along with the relative-phase at the central wavelengths, are shown. It is evident that the phase gradients of the superlattice structure and the double-nonlinearity crystal are lower by more than one order of magnitude, compared with that of the cascaded crystals.

### Spatial characteristics

The biphoton angular spectrum described by equation (11) corresponds to a spatial distribution in the exit plane of the crystal (*z* = 0)^56^ given by

13 $$\begin{array}{ccc}{P}_{{\rm{H}},{\rm{V}}}({{\bf{x}}}_{1},{{\bf{x}}}_{2}) & = & \int d{\omega }_{1}d{\omega }_{2}| \int d{{\bf{q}}}_{1}d{{\bf{q}}}_{2}{{\rm{\Phi }}}_{{\rm{H}},{\rm{V}}}({\omega }_{1},{\omega }_{2};{{\bf{q}}}_{1},{{\bf{q}}}_{2})\\  &  & {\times G({\omega }_{1}-{\omega }_{1}^{0})G({\omega }_{2}-{\omega }_{2}^{0}){{\rm{e}}}^{i({{\bf{q}}}_{1}.{{\bf{x}}}_{1}+{{\bf{q}}}_{2}.{{\bf{x}}}_{2})}| }^{2}.\end{array}$$

**Figure 5:**
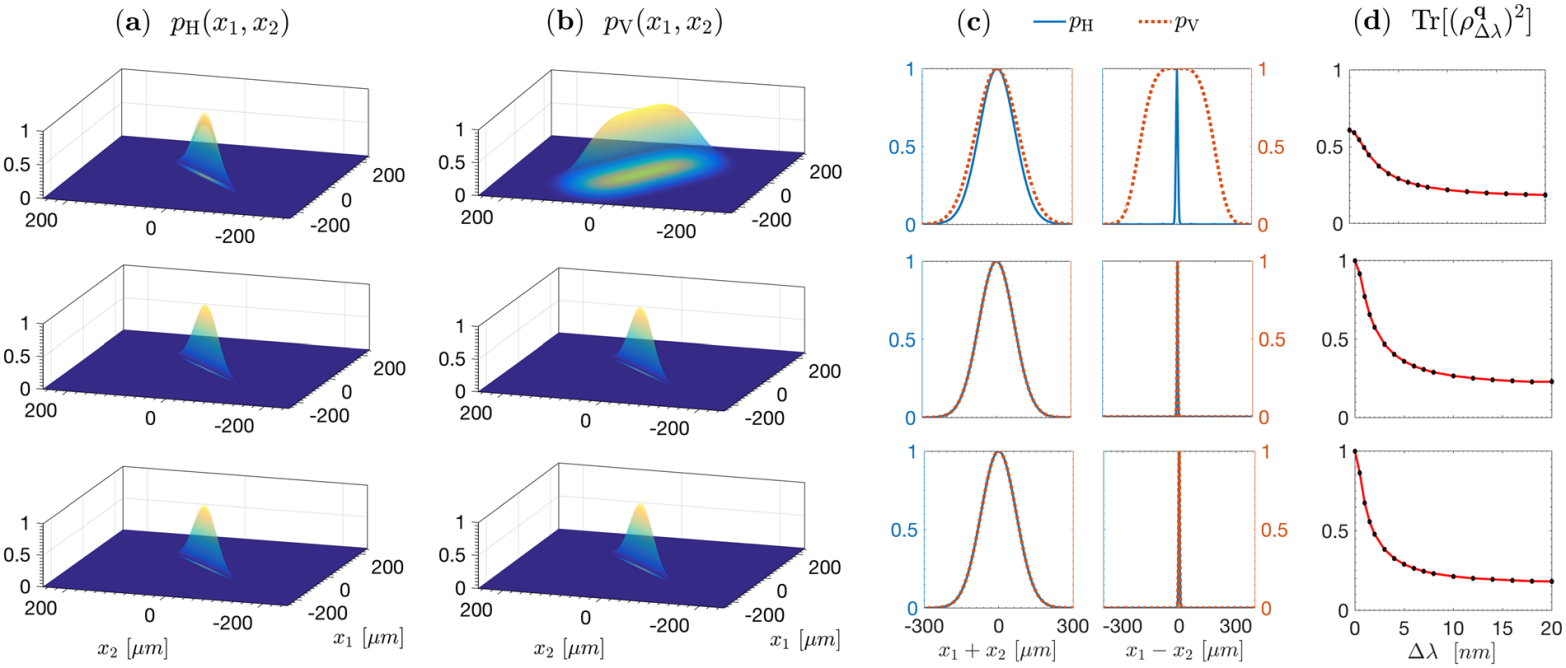
Comparison of the spatial characteristics of biphotons emitted by three LiNbO_3_ structures—cascaded (top), double-nonlinearity (middle), and superlattice (bottom)— of 5-mm overall length, excited by a 532-nm, 133-fs pulsed pump focused to a 50-μ﻿m waist. The downconversion is nondegenerate with signal and idler photons centered at 810 nm and 1550 nm, and noncollinear with the respective central emission angles 0.5° and 0.96° in free space, respectively. Ultra-wide band spectral filters (70-nm full width at half maximum) are used. (**a**) The biphoton probability distribution *P*_H_(**x**_1_, **x**_2_) for the HH component at the crystal exit plane *z* = 0 shown here at points on the *x* axis as a function *p*_H_(*x*_1_, *x*_2_) = *P*_H_(*x*_1_, 0, *x*_2_, 0). (**b**) Same as (**a**) but for the VV component. (**c**) Projections of *p*_H_(*x*_1_, *x*_2_) and *p*_V_(*x*_1_, *x*_2_) along the diagonal and off-diagonal directions as functions of (*x*_1_ + *x*_2_) and (*x*_1_ − *x*_2_), normalized to their respective maximum values. For the cascaded crystals (first row), the high quadratic relative-phase function in (*q*_1*x*_ − *q*_2*x*_) axis in Fig. 4 introduces a magnifying distortion to the joint spatial correlations along (*x*_1_ − *x*_2_) axis. The superlattice and double-nonlinearity crystal have nearly perfect spatial indistinguishability. (**d**) Spatial purity ${\rm{Tr}}[{({\rho }_{{\rm{\Delta }}\lambda }^{{\bf{q}}})}^{2}]$ as a function of the spectral filter bandwidth Δ*λ* (full width at half maximum). At Δ*λ* = 0, the purity is unity for both the DN and SL structures, since the distinguishing frequency information is absent. As Δ*λ* increases, the spatial purity is reduced, indicating that the spectral degree of freedom plays an increasing distinguishing role, together with the polarization degree of freedom.

This distribution, which may be observed by measuring photon coincidence at pairs of positions in an image of the crystal’s exit plane, is depicted in Fig. 5 for points on the *x* axis. As expected, the relative-phase gradient in the angular (transverse momentum) domain has a significant effect on the distribution in the conjugate position domain. This relative phase exhibits a strong quadratic dependence on (*q*_1*x*_ − *q*_2*x*_) as depicted in Fig. 4d, which corresponds to a quadratic phase function of (*x*_1_ − *x*_2_) in the spatial domain, introducing distortion much like Fresnel diffraction along the position difference (*x*_1_ − *x*_2_).

For the cascaded-crystals, relative to the $|HH\rangle$ pairs (which have coincidences more likely happen near momentum-conserved modes), the $|VV\rangle$ pairs can have coincidences quite far from such a tight mapping. By contrast, it is obvious how the two-photon wavepackets of the superlattice and double-nonlinearity structures are well indistinguishable in space, owing to the low phase gradient compared with the cascaded crystals.

Figure 4a,b exhibit the anti-correlation nature of the two-photon directions in the far field originating from the phase matching in the nonlinear domains. In the conjugate near-field zone, Fig. 5a,b display the correlation in the two-photon positions because of their co-located birth from a downconverted pump photon. Here, the $|VV\rangle$ biphotons produced by the cascaded crystal show weaker positional correlations, which denotes the migration of the transverse-spatial entanglement out of the wavefunction modulus to the associated phase in the coordinate space^57^. The migration-to-phase process is then reversed after arbitrary propagation^58^ so that correlations are restored in the far field, as Fig. 4b shows.

We now consider entanglement in the spatial degree of freedom and its possible reduction as a result of distinguishing information from the polarization and frequency degrees of freedom. Several measures can be adopted, including Schmidt analysis of the biphoton wavefunction^59, 60^. We use here the purity of the spatial biphoton state, which is obtained by tracing out other degrees of freedom (see Methods):

14 $$\begin{array}{rcl}{\rm{Tr}}[{({\rho }_{{\rm{\Delta }}\lambda }^{{\bf{q}}})}^{2}] & = & \int d{\omega }_{1}d{\omega }_{2}d{{\bf{q}}}_{1}d{{\bf{q}}}_{2}d{\omega }_{1}^{\prime}d{\omega }_{2}^{\prime}d{{\bf{q}}}_{1}^{\prime}d{{\bf{q}}}_{2}^{\prime}\\  &  & \times \,{[G({\omega }_{1}-{\omega }_{1}^{0})G({\omega }_{2}-{\omega }_{2}^{0})]}^{2}[{{\rm{\Phi }}}_{{\rm{H}}}({\omega }_{1},{\omega }_{2};{{\bf{q}}}_{1},{{\bf{q}}}_{2}){{\rm{\Phi }}}_{{\rm{H}}}^{\ast }({\omega }_{1},{\omega }_{2};{{\bf{q}}}_{1}^{\prime},{{\bf{q}}}_{2}^{\prime})\\  &  & +\,{{\rm{\Phi }}}_{{\rm{V}}}({\omega }_{1},{\omega }_{2};{{\bf{q}}}_{1},{{\bf{q}}}_{2}){{\rm{\Phi }}}_{{\rm{V}}}^{\ast }({\omega }_{1},{\omega }_{2};{{\bf{q}}}_{1}^{\prime},{{\bf{q}}}_{2}^{\prime})]\\  &  & \times \,{[G({\omega }_{1}^{\prime}-{\omega }_{1}^{0})G({\omega }_{2}^{\prime}-{\omega }_{2}^{0})]}^{2}[{{\rm{\Phi }}}_{{\rm{H}}}({\omega }_{1}^{\prime},{\omega }_{2}^{\prime};{{\bf{q}}}_{1}^{\prime},{{\bf{q}}}_{2}^{\prime}){{\rm{\Phi }}}_{{\rm{H}}}^{\ast }({\omega }_{1}^{\prime},{\omega }_{2}^{\prime};{{\bf{q}}}_{1},{{\bf{q}}}_{2})\\  &  & +\,{{\rm{\Phi }}}_{{\rm{V}}}({\omega }_{1}^{\prime},{\omega }_{2}^{\prime};{{\bf{q}}}_{1}^{\prime},{{\bf{q}}}_{2}^{\prime}){{\rm{\Phi }}}_{{\rm{V}}}^{\ast }({\omega }_{1}^{\prime},{\omega }_{2}^{\prime};{{\bf{q}}}_{1},{{\bf{q}}}_{2})]\mathrm{.}\end{array}$$

Figure 5d depicts ${\rm{Tr}}[{({\rho }_{{\rm{\Delta }}\lambda }^{{\bf{q}}})}^{2}]$ as a function of the detection bandwidth. For monochromatic detection, the frequency information is suppressed so that the distinguishability is only due to polarization. Note that emission from the cascaded crystals exhibits a strong effect of polarization on the spatial degree of freedom. The superlattice and double-nonlinearity structures avoid such effect by virtue of the interleaved and co-located downconversion, respectively, producing $|HH\rangle$ and $|VV\rangle$ biphotons. However, the spectral information appears to be a more dominant distinguishing effect for wider detection bandwidth. This effect originates from the inherent coupling between the spectral and spatial degrees of freedom in the SPDC two-photon state^55^.

### Spectral characteristics

**Figure 6:**
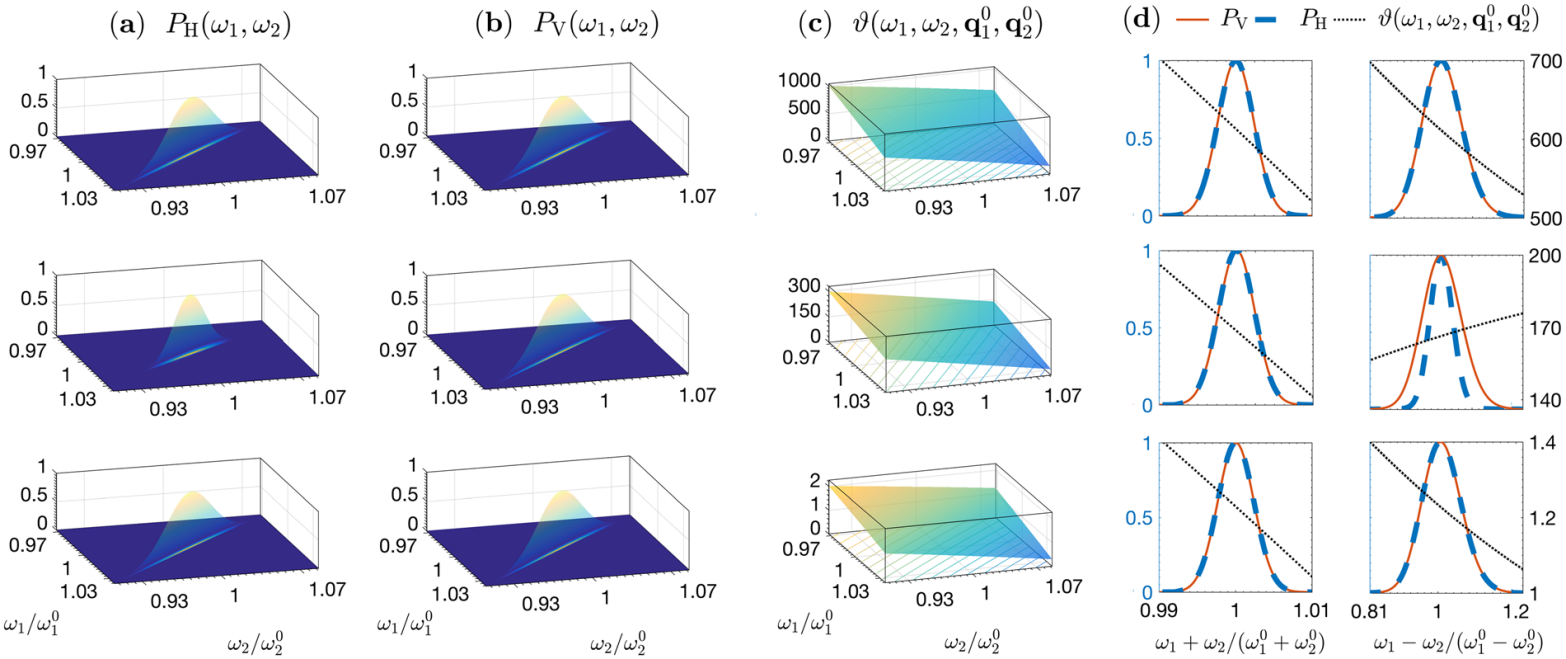
Comparison of the spectral characteristics of biphotons emitted by three LiNbO_3_ structures—cascaded crystals (top), double-nonlinearity (middle), and superlattice (bottom)— of 5-mm overall length, excited by a 532-nm, 133-fs pulsed pump focused to a 50-μ﻿m waist. The emitted light is collected through two Gaussian apertures placed in the focal planes of two identical 2-f systems of 15-cm focal lengths and with ${\sigma }_{{q}_{1}}={\sigma }_{{q}_{2}}=60$ mm^−1^, corresponding to apertures widths of 1.2 mm (16-mrad collection angle) and 2.2 mm (29-mrad collection angle) for the signal and idler, respectively. Due to the phase-matching condition, the signal and idler bandwidth set by the apertures is ~750 nm. (**a**) The biphoton probability distribution *P*_H_(*ω*_1_, *ω*_2_) for the HH component. (**b**) Same as (**a**) but for the VV component. (**c**) Relative phase *ϑ* [rad] between the superposition weights of the |*HH*〉 and |*VV*〉 states as a function of *ω*_1_ and *ω*_2_. An overall phase offset is subtracted for clarity. (**d**) Projections of *P*_H_(*ω*_1_, *ω*_2_) and *P*_V_(*ω*_1_, *ω*_2_) along the diagonal and off-diagonal directions as functions of (*ω*_1_ + *ω*_2_) and (*ω*_1_ − *ω*_2_), normalized to their respective maximum values.

The spectral characteristics of the photon pairs are highlighted by integrating out the spatial dependence of the state functions over two solid angles surrounding the central vectors ${{\bf{q}}}_{\mathrm{1,2}}^{0}$ (corresponding to central emission angles ${\theta }_{\mathrm{1,2}}^{0}\approx c|{{\bf{q}}}_{\mathrm{1,2}}^{0}|/{\omega }_{\mathrm{1,2}}$). In practice, this may be implemented by collecting the light through two apertures in the focal plane of a 2-f Fourier-transforming systems mapping emission directions to detection positions. The resultant joint probability density functions are

15 $${P}_{{\rm{H}},{\rm{V}}}({\omega }_{1},{\omega }_{2})=\int d{{\bf{q}}}_{1}d{{\bf{q}}}_{2}{|{{\rm{\Phi }}}_{{\rm{H}},{\rm{V}}}({\omega }_{1},{\omega }_{2};{{\bf{q}}}_{1},{{\bf{q}}}_{2}){F}_{1}({{\bf{q}}}_{1}-{{\bf{q}}}_{1}^{0}){F}_{2}({{\bf{q}}}_{2}-{{\bf{q}}}_{2}^{0})|}^{2},$$

where ${F}_{\mathrm{1,2}}({\bf{q}})=(\mathrm{1/}\sqrt[4]{\pi {\sigma }_{q\mathrm{1,2}}^{2}})\exp (-{|{\bf{q}}|}^{2}\mathrm{/2}{\sigma }_{q\mathrm{1,2}}^{2})$ are aperture functions, taken to be Gaussian with widths ${\sigma }_{{q}_{\mathrm{1,2}}}$, assumed to be fixed within the bandwidths of interest. These spectral distributions are shown in Fig. 6.

It is useful here to think about the two-photon wavefunctions as being formulated due to two contributions; one of them is by the spatial-spectral modes of the interacting fields, while the other is for the geometry of the illuminated structure itself^46^. Along our comparisons, the contribution due to the interacting fields is always the same while the geometries of the nonlinear structures lead to the different outcomes.

Also shown in Fig. 6 is the distribution of the relative phase between *P*_V_ and *P*_H_ evaluated at the central directions ${{\bf{q}}}_{\mathrm{1,2}}^{0}$. Here too, the gradient of the relative phase is significantly smaller for the superlattice structure in comparison with double-nonlinearity and cascaded structures. Since the relative phase is approximately linear within the range for which the magnitudes of the functions are significant, this effect amounts to a corresponding shift in the time domain, as we will see next.

### Temporal characteristics

**Figure 7:**
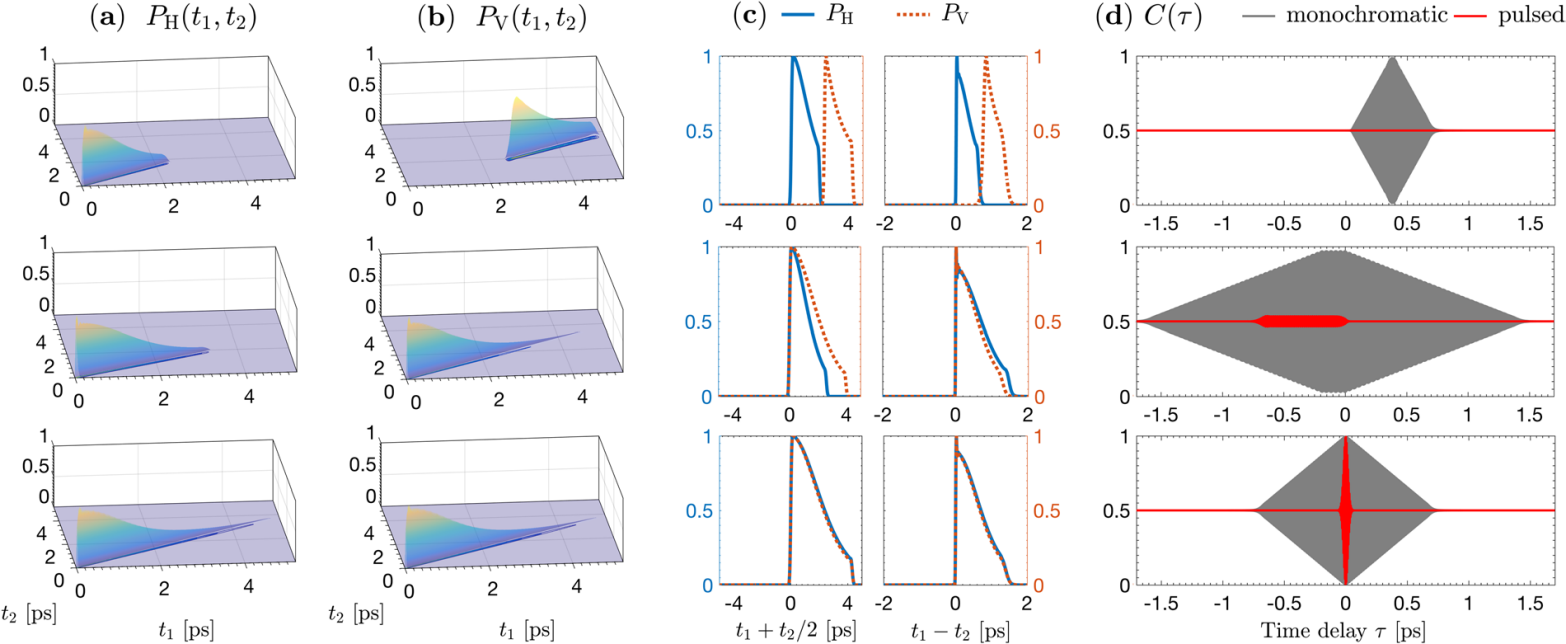
Comparison of the temporal characteristics of biphotons emitted by three LiNbO_3_ structures—cascaded (top), double-nonlinearity (middle), and superlattice (bottom)—of 5-mm overall length, excited by a 532-nm, 133-fs pulsed pump focused to a 50-μ﻿m waist. The emitted light is collected through two Gaussian apertures placed in the focal planes of two identical 2-f systems of 15-cm focal lengths and with ${\sigma }_{{q}_{1}}={\sigma }_{{q}_{2}}=60$ mm^−1^, corresponding to apertures widths of 1.2 mm (16-mrad collection angle) and 2.2 mm (29-mrad collection angle) for the signal and idler, respectively. Due to the phase-matching condition, the signal and idler bandwidth set by the apertures is ~750 nm. (**a**) The biphoton probability distribution *P*_H_(*t*_1_, *t*_2_) for the HH component. (**b**) Same as (**a**) but for the VV component. (**c**) Projections of *P*_H_(*t*_1_, *t*_2_) and *P*_V_(*t*_1_, *t*_2_) along the diagonal and off-diagonal directions as functions of the average arrival time (*t*_1_ + *t*_2_)/2 and the relative delay (*t*_1_ − *t*_2_) between the signal and idler, normalized to their respective maximum values. The relative time delay is due to first-order dispersion, while dispersion of higher order contribute to the biphoton wavepacket distortion. (**d**) Normalized coincidence rate *C*(*τ*), after HOM interferometer, expected for a monochromatic (gray line) and a 133-fs pulsed (red line) pump beam. Here, the apertures are pinholes placed in the central emission directions. The sinusoidal oscillation within the dip envelope is at frequency (*ω*_1_ − *ω*_2_).

In the time domain, the wavepackets of the HH and VV biphotons are described by the probability densities

16 $${P}_{{\rm{H}},{\rm{V}}}({t}_{1},{t}_{2})=\int d{{\bf{q}}}_{1}d{{\bf{q}}}_{2}{| \int d{\omega }_{1}d{\omega }_{2}{{\rm{\Phi }}}_{{\rm{H}},{\rm{V}}}({\omega }_{1},{\omega }_{2};{{\bf{q}}}_{1},{{\bf{q}}}_{2}){F}_{1}({{\bf{q}}}_{1}-{{\bf{q}}}_{1}^{0}){F}_{2}({{\bf{q}}}_{2}-{{\bf{q}}}_{2}^{0}){{\rm{e}}}^{-i({\omega }_{1}{t}_{1}+{\omega }_{2}{t}_{2})}| }^{2},$$

where *t*_1_ and *t*_2_ are the times at which the signal and idler photons cross the structure’s exit plane (*z* = 0)^56^. These functions are displayed in Fig. 7 along with their projections along the time difference (*t*_1_ − *t*_2_) and the time average $\frac{1}{2}({t}_{1}+{t}_{2})$. For all three structures, both the HH and the VV biphotons are narrow functions of the time difference, indicating time correlation (in the subpicosecond regime) and broad functions of the time average, indicating temporal entanglement. The breadth along the time average is constrained by the combined effect of the pump pulse width, the group velocity mismatch between interacting waves, and the limited length of interaction domain.

For the cascaded-crystals source, the two-photon wavepackets emerge with an average delay between the two signals and between the two idlers of the $|HH\rangle$ and $|VV\rangle$ polarization are [*u* ^*o*^(*ω*_*p*_) − *u* ^*o*^(*ω*_1,2_)]*L*/2 ≈ {1.9 ps, 2.7 ps}, where *u* ^*o*^(*ω*_*j*_) is the reciprocal group velocity of ordinary-polarized light at angular frequency *ω*_*j*_. Also because of dispersion, the time delay between the faster idler and the slower signal belonging to the same pair conveys distinguishing information about the crystal of origin. The average of this dispersive delay is [*u* ^*o*^(*ω*_1_) − *u* ^*o*^(*ω*_2_)]*L*/2 ≈ 760 fs. The two types of delay are represented in the projections of Fig. 7c. While the distinguishability in the mean arrival time $\frac{1}{2}({t}_{1}+{t}_{2})$ is mainly due to the group-velocity mismatch between the pump and downconverted photons, that in the time difference (*t*_1_ − *t*_2_) is due to the group-velocity mismatch between the nondegenerate signal and idler photons. Such time delay renders the two superposed terms of the state entirely distinguishable, so that the *entangled area* ^26^ effectively shrinks to zero. It is required that these relative delays be much less than the coherence time of the pump field in order to suppress the temporal distinguishing information.

For the double-nonlinearity crystal source, the delay between the two signals and two idlers of the $|HH\rangle$ and $|VV\rangle$ possibilities depends on their birth position, with the average time delay being [*u* ^*o*^(*ω*_1,2_) − *u* ^*e*^(*ω*_1,2_)]*L*/2 ≈ {0.73 ps, 0.64 ps}, where *u* ^*e*^(*ω*_*j*_) is the reciprocal group velocity for the extraordinary polarization at angular frequency *ω*_*j*_. The distinguishing dispersive delay between the faster idler and the slower signal belonging to the same pair is {[*u* ^*o*^(*ω*_1_) − *u* ^*o*^(*ω*_2_)] − [*u* ^*e*^(*ω*_1_) − *u* ^*e*^(*ω*_2_)]}*L*/2 ≈ 88 fs on average. These delays introduce partial distinguishability as there is always a region emitting temporally indistinguishable pairs at the end of the DN crystal.

For the superlattice source, emission has double the coherence time of the cascaded crystals due to the uncertainty regarding the emission origin along the whole structure length. The signals and the idlers of the $|HH\rangle$ and $|VV\rangle$ possibilities emerge with an average delay of [*u* ^*o*^(*ω*_*p*_) − *u* ^*o*^(*ω*_1,2_)]*d*/2 ≈ {3.9 fs, 5.4 fs}. The average dispersive delay between the faster idler and the slower signal of the same pair is [*u* ^*o*^(*ω*_1_) − *u* ^*o*^(*ω*_2_)]*d*/2 ≈ 1.5 fs. Both types of delay are proportional to the two-layer thickness *d* instead of the total length *L* of the structure, as in the case of the other two structures (see space-time diagrams in Fig. 1b).

Temporal (spectral) entanglement is often assessed via Hong-Ou-Mandel (HOM) interferometry^61^. For degenerate SPDC photons, the spectral symmetry of the joint two-photon amplitude, i.e., Φ(*ω*_1_, *ω*_2_) = Φ(*ω*_2_, *ω*_1_), leads to perfect visibility of HOM interference. For nondegenerate photons, a HOM-type two-color interferometer has been implemented^62^. For our nondegenerate system, which features spectral, spatial, and polarization degrees of freedom, we use light emitted in four directions falling on two rings within the emission cone and centered at modes ${{\rm{M}}}_{1}=({{\bf{q}}}_{1}^{0},{\omega }_{1}^{0})$ and ${{\rm{M}}}_{2}=({{\bf{q}}}_{2}^{0},{\omega }_{2}^{0})$ and their matching modes ${{\rm{M}}}_{1}^{\prime}=(-{{\bf{q}}}_{1}^{0},{\omega }_{2}^{0})$ and ${{\rm{M}}}_{2}^{\prime}=(-{{\bf{q}}}_{2}^{0},{\omega }_{1}^{0})$, where ${\omega }_{1}^{0}$ and ${\omega }_{2}^{0}$ are the central angular frequencies. The polarization of each pair of matched modes is either HH or VV with the superposition weights ${{\rm{\Phi }}}_{{\rm{H}}}^{{\rm{o}}}({\omega }_{1},{\omega }_{2})$ and ${{\rm{\Phi }}}_{{\rm{V}}}^{{\rm{o}}}({\omega }_{1},{\omega }_{2})$ for the $({{\rm{M}}}_{1},{{\rm{M}}}_{1}^{\prime})$ pair, and ${{\rm{\Phi }}}_{{\rm{H}}}^{{\rm{o}}}({\omega }_{2},{\omega }_{1})$ and ${{\rm{\Phi }}}_{{\rm{V}}}^{{\rm{o}}}({\omega }_{2},{\omega }_{1})$ for the $({{\rm{M}}}_{2},{{\rm{M}}}_{2}^{\prime})$ pair, where ${{\rm{\Phi }}}_{{\rm{H}},{\rm{V}}}^{{\rm{o}}}({\omega }_{1},{\omega }_{2})={{\rm{\Phi }}}_{{\rm{H}},{\rm{V}}}({\omega }_{1},{\omega }_{2};{{\bf{q}}}_{1}^{0},-{{\bf{q}}}_{1}^{0})$ and ${{\rm{\Phi }}}_{{\rm{H}},{\rm{V}}}^{{\rm{o}}}({\omega }_{2},{\omega }_{1})={{\rm{\Phi }}}_{{\rm{H}},{\rm{V}}}({\omega }_{2},{\omega }_{1};{{\bf{q}}}_{2}^{0},-{{\bf{q}}}_{2}^{0})$. After rotating the polarization of (M_2_, ${{\rm{M}}}_{2}^{\prime}$), pair by 90^o^ using half-wave plates, the four modes are transmitted into the two ports of a beam splitter, so that each of the HH and VV components can interfere (see Methods).

With a time delay *τ* introduced at one port of the beam splitter, the probability of coincidence, i.e., detecting one photon at each port, is given by

17 $$\begin{array}{rcl}p\mathrm{(1,}\,\mathrm{1)} & = & \frac{1}{4}\int {|{{\rm{e}}}^{-i{\omega }_{1}\tau }{{\rm{\Phi }}}_{{\rm{H}}}^{{\rm{o}}}({\omega }_{1},{\omega }_{2})-{{\rm{e}}}^{-i{\omega }_{2}\tau }{{\rm{\Phi }}}_{{\rm{V}}}^{{\rm{o}}}({\omega }_{2},{\omega }_{1})|}^{2}\\  &  & +{|{{\rm{e}}}^{-i{\omega }_{1}\tau }{{\rm{\Phi }}}_{{\rm{V}}}^{{\rm{o}}}({\omega }_{1},{\omega }_{2})-{{\rm{e}}}^{-i{\omega }_{2}\tau }{{\rm{\Phi }}}_{{\rm{H}}}^{{\rm{o}}}({\omega }_{2},{\omega }_{1})|}^{2}d{\omega }_{1}d{\omega }_{2},\end{array}$$

Therefore, the normalized coincidence rate *C*(*τ*) is given by

18 $$C(\tau )=\frac{1}{2}-\frac{\Re {\mathfrak{e}}[{C}_{{\rm{HV}}}(\tau )]}{{C}_{{\rm{HH}}}\mathrm{(0)}+{C}_{{\rm{VV}}}\mathrm{(0)}},$$

where

19 $${C}_{{\rm{H}}{\rm{V}}}(\tau )=\int d{\omega }_{1}d{\omega }_{2}{{\rm{\Phi }}}_{{\rm{H}}}^{{\rm{o}}}({\omega }_{1},{\omega }_{2}){{\rm{\Phi }}}_{{\rm{V}}}^{{{\rm{o}}}^{\ast }}({\omega }_{2},{\omega }_{1}){{\rm{e}}}^{i({\omega }_{2}-{\omega }_{1})\tau },$$

and $\Re {\mathfrak{e}}$ denotes the real part. The relative delay *τ* corresponds to translating one of the wavefunctions in Fig. 7a,b, with respect to the other, introducing a time difference (*t*_1_ − *t*_2_) = 2*τ*. The normalized coincidence rate *C*(*τ*) measures their degree of overlap. Note that the wavepackets are indistinguishable in terms of the mean time $\frac{1}{2}({t}_{1}+{t}_{2})$ in case of monochromatic pumping. Figure 7d is a plot of *C*(*τ*) for the three SPDC sources in two cases: a monochromatic pump, and a pulsed pump producing a train of 133-fs pulses. Since the signal and idler are highly nondegenerate, the familiar HOM dips are modulated by sinusoidal oscillations at the beat frequency (*ω*_1_ − *ω*_2_).

For the CC and DN sources, the HOM dips are time-shifted from *τ* = 0. This time offset represents the required temporal compensation, which is generally introduced by use of birefringent element(s). The dip depth (visibility) is a measure of the degree of temporal indistinguishability after optimal compensation. For the DN crystal, it is impossible to realize full visibility, even with a monochromatic pump, due to the discrepancy in the temporal biphoton wavepacket from type-0 and type-I interactions, as highlighted in Fig. 7a,b. When the $|HH\rangle$ and $|VV\rangle$ possibilities are due to type-0 interaction (CC and superlattice cases), the dip width is the wavepacket length (coherence time) of the downconverted biphoton. Note that the dip width for the superlattice source is twice that for the cascaded crystals. However, when a pulsed pump is used, only the superlattice source, which features continuous group-velocity matching (see Fig. 1), can realize nearly perfect visibility without temporal compensation.

## Discussion

We have introduced a new superlattice structure with unit cell composed of two orthogonal nonlinear thin layers, one producing horizontally polarized biphotons and the other producing vertically polarized biphotons. QPM is integrated into the same superlattice by poling the periodic layers in alternating orthogonal directions. The SPDC biphotons are emitted in a maximally entangled superposition of the $|HH\rangle$ and $|VV\rangle$ states over an emission cone significantly wider than for other sources, and with a broader spectral width. The distinguishing information is limited by the thickness of the layers. Within the emission cone, the magnitudes of the quantum state superposition weights are almost identical and their spatial-spectral relative-phase gradient is smaller by several orders of magnitude compared to other sources, depending on the thickness of the layers and their number. Spectral and angular (transverse-momentum) entanglement are also preserved, so that the overall quantum state is hyperentangled.

The higher degree of spatial and temporal indistinguishability of the biphotons generated by the superlattice structure enables tight focusing of the pump into a long crystal, even in the noncollinear configuration — a combination that severely limits biphoton flux rates in ordinary sources. The superlattice SPDC source also supports the use of a pump with low coherence time and enables all-over-the-cone two-photon collection, all without the need for compensation.

The parameters and/or configuration of the superlattice structure presented in this paper may be modified in several ways to obtain quantum states with different features. For example, instead of using third-order QPM, which leads to a delay of about {3.9 fs, 5.4 fs} between the $|HH\rangle$ and $|VV\rangle$ possibilities, in a chirped structure with first-order QPM this delay may be compressed to less than a monocycle of light. This offers a hyperentangled version of the original proposal by Harris^43^ to generate temporally entangled photon pairs correlated to less than one cycle of light. A superlattice *chirp-and-compress* design will generate noncollinear ultra-broadband biphotons with no need for large chirping^44, 45^ and no need for self-compensation to eliminate temporal distinguishability, and hence no need for an additional time-delay element.

The superlattice source can be readily adapted to produce a tailored two-photon polarization state similar to the one first suggested by Klyshko^63^. By use of two or three nonlinear layers per each set of the sequence an arbitrary biphoton polarization state $\alpha |{H}_{1}{H}_{2}\rangle +\beta |{H}_{1}{V}_{2}\rangle +\gamma |{V}_{1}{H}_{2}\rangle +\delta |{V}_{1}{V}_{2}\rangle$ may be created with angular biphoton spectrum controlled by the geometry of the layered structure. While preserving the spectral and momentum entanglement, the output polarization state can *surf* the *S* ^4^ sphere in a five-dimensional space. The feasibility to create such arbitrary polarization state is of fundamental importance as there is no way to generate it by the use of polarization transformers (characterized by two parameters) to move between two arbitrary points on the *S* ^4^ sphere.

The fabrication of the proposed superlattice structure will be challenging. The implementation of interleaved periodic poling in alternating orthogonal directions will require the development of new poling processes. Fabrication of a periodic thin-layered structure that alternatingly emits biphotons of orthogonal polarization without orthogonal poling is feasible by engineering the grating periods. However, such structure will require additional elements to suppress temporal walkoff.

## Methods

### Two-photon amplitude

In our analysis, we assume weak scattering so that the SPDC process remains spontaneous and the pump field is undepleted. We also neglect reflected waves at all interfaces and assume that the nonlinear layers have infinite transverse extent so that all fields do not interact with the transverse boundaries. The two-photon wavefunctions of the hyperentangled state (1) can be expressed as

20 $$\begin{array}{rcl}{{\rm{\Phi }}}_{{\rm{H}}}({\omega }_{1},{\omega }_{2};{{\bf{q}}}_{1},{{\bf{q}}}_{2}) & = & {\sigma }_{{\rm{H}}}{\chi }^{\mathrm{(2)}}{A}_{p}({\omega }_{1}+{\omega }_{2};{{\bf{q}}}_{1}+{{\bf{q}}}_{2}){\int }_{-L}^{0}dz\,{g}_{{\rm{H}}}(z){e}^{i{\rm{\Delta }}{\varphi }_{{\rm{H}}}({\omega }_{1},{\omega }_{2};{{\bf{q}}}_{1},{{\bf{q}}}_{2},z)},\\ {{\rm{\Phi }}}_{{\rm{V}}}({\omega }_{1},{\omega }_{2};{{\bf{q}}}_{1},{{\bf{q}}}_{2}) & = & {\sigma }_{{\rm{V}}}{\chi }^{\mathrm{(2)}}{A}_{p}({\omega }_{1}+{\omega }_{2};{{\bf{q}}}_{1}+{{\bf{q}}}_{2}){\int }_{-L}^{0}dz\,{g}_{{\rm{V}}}(z)\,{e}^{i{\rm{\Delta }}{\varphi }_{{\rm{V}}}({\omega }_{1},{\omega }_{2};{{\bf{q}}}_{1},{{\bf{q}}}_{2},z)},\end{array}$$

where *g*_H,V_(*z*) are periodic rectangular functions (see Fig. 1) corresponding to the modulations of the H and V layers, respectively, the accumulated longitudinal phase mismatch ${\rm{\Delta }}{\varphi }_{H,V}({\omega }_{1},{\omega }_{2};{{\bf{q}}}_{1},{{\bf{q}}}_{2},z)={\int }_{0}^{z}{\rm{\Delta }}\kappa (z^{\prime} \mathrm{)\ }dz^{\prime}$ can be expressed as

21 $$\begin{array}{rcl}{\rm{\Delta }}{\varphi }_{{\rm{H}}}({\omega }_{1},{\omega }_{2};{{\bf{q}}}_{1},{{\bf{q}}}_{2},z) & = & {\rm{\Delta }}{\kappa }_{{\rm{H}}}^{e}z+({\rm{\Delta }}{\kappa }_{H}^{e}-{\rm{\Delta }}{\kappa }^{o})(\frac{M}{2}-\ell ){d}_{{\rm{V}}}\\ {\rm{\Delta }}{\varphi }_{{\rm{V}}}({\omega }_{1},{\omega }_{2};{{\bf{q}}}_{1},{{\bf{q}}}_{2},z) & = & {\rm{\Delta }}{\kappa }_{{\rm{V}}}^{e}z+({\rm{\Delta }}{\kappa }_{V}^{e}-{\rm{\Delta }}{\kappa }^{o})(\frac{M}{2}-\ell +1){d}_{{\rm{H}}}\mathrm{.}\end{array}$$

Here $\ell$ is the number of the two-layer sets, counted from the entrance plane *z* = −*L* (to the right). The output state is thus a coherent superposition of all two-photon amplitudes along the structure layers which can be expressed as

22 $$\begin{array}{rcl}{{\rm{\Phi }}}_{{\rm{H}}}({\omega }_{1},{\omega }_{2};{{\bf{q}}}_{1},{{\bf{q}}}_{2}) & = & {\sigma }_{{\rm{H}}}{\chi }^{\mathrm{(2)}}{A}_{p}({\omega }_{1}+{\omega }_{2};{{\bf{q}}}_{1}+{{\bf{q}}}_{2}){{\sum }_{\ell =1}}^{\frac{M}{2}}{(-1)}^{\ell -1}{\int }_{-L+\ell d-{d}_{{\rm{H}}}}^{-L+\ell d}dz\,{e}^{i{\rm{\Delta }}{\varphi }_{{\rm{H}}}(z)},\\ {{\rm{\Phi }}}_{{\rm{V}}}({\omega }_{1},{\omega }_{2};{{\bf{q}}}_{1},{{\bf{q}}}_{2}) & = & {\sigma }_{{\rm{V}}}{\chi }^{\mathrm{(2)}}{A}_{p}({\omega }_{1}+{\omega }_{2};{{\bf{q}}}_{1}+{{\bf{q}}}_{2}){{\sum }_{\ell =1}}^{\frac{M}{2}}{(-1)}^{\ell -1}{\int }_{-L+(\ell -1)d}^{-L+\ell d-{d}_{{\rm{H}}}}dz\,{e}^{i{\rm{\Delta }}{\varphi }_{{\rm{V}}}(z)},\end{array}$$

and can be rewritten as

23 $$\begin{array}{ccc}{{\rm{\Phi }}}_{{\rm{H}}}({\omega }_{1},{\omega }_{2};{{\bf{q}}}_{1},{{\bf{q}}}_{2}) & = & {\sigma }_{{\rm{H}}}{\chi }^{(2)}{d}_{{\rm{H}}}{A}_{p}({\omega }_{1}+{\omega }_{2};{{\bf{q}}}_{1}+{{\bf{q}}}_{2})\,{\rm{s}}{\rm{i}}{\rm{n}}{\rm{c}}(\frac{{\rm{\Delta }}{\kappa }_{{\rm{H}}}^{e}{d}_{{\rm{H}}}}{2})\\  &  & \times {e}^{i[(1-\frac{M}{2})({\rm{\Delta }}{\kappa }_{{\rm{H}}}^{e}{d}_{{\rm{H}}}+{\rm{\Delta }}{\kappa }^{o}{d}_{{\rm{V}}})-\frac{{\rm{\Delta }}{\kappa }_{{\rm{H}}}^{e}{d}_{{\rm{H}}}}{2}]}{\sum }_{\ell =1}^{\frac{M}{2}}{e}^{i(\ell -1)({\rm{\Delta }}{\kappa }^{o}{d}_{{\rm{V}}}+{\rm{\Delta }}{\kappa }_{{\rm{H}}}^{e}{d}_{{\rm{H}}}-\mu \pi )},\\ {{\rm{\Phi }}}_{{\rm{V}}}({\omega }_{1},{\omega }_{2};{{\bf{q}}}_{1},{{\bf{q}}}_{2}) & = & {\sigma }_{{\rm{V}}}{\chi }^{(2)}{d}_{{\rm{V}}}{A}_{p}({\omega }_{1}+{\omega }_{2};{{\bf{q}}}_{1}+{{\bf{q}}}_{2})\,{\rm{s}}{\rm{i}}{\rm{n}}{\rm{c}}\,(\frac{{\rm{\Delta }}{\kappa }_{{\rm{V}}}^{e}{d}_{{\rm{V}}}}{2})\\  &  & \times {e}^{-i[\frac{M}{2}({\rm{\Delta }}{\kappa }_{{\rm{V}}}^{e}{d}_{{\rm{V}}}+{\rm{\Delta }}{\kappa }^{o}{d}_{{\rm{H}}})-\frac{{\rm{\Delta }}{\kappa }_{{\rm{V}}}^{e}{d}_{{\rm{V}}}}{2}]}{\sum }_{\ell =1}^{\frac{M}{2}}{e}^{i(\ell -1)({\rm{\Delta }}{\kappa }^{o}{d}_{{\rm{H}}}+{\rm{\Delta }}{\kappa }_{{\rm{V}}}^{e}{d}_{{\rm{V}}}-\mu \pi )},\end{array}$$

It is then straightforward to determine the biphoton wavefunctions expressed in equation (2).

### Reduced density matrix

The density matrix of the pure state (1) can be written as

24 $$\begin{array}{ccc}\rho  & = & \int d{\omega }_{1}d{\omega }_{2}d{{\bf{q}}}_{1}d{{\bf{q}}}_{2}d{\omega }_{1}^{\prime}d{\omega }_{2}^{\prime}d{{\bf{q}}}_{1}^{\prime}d{{\bf{q}}}_{2}^{\prime}\\  &  & \times |{\omega }_{1};{{\bf{q}}}_{1}\rangle |{\omega }_{2};{{\bf{q}}}_{2}\rangle {\sum }_{{\rm{a}},{{\rm{a}}}^{\prime}=\{{\rm{H}},{\rm{V}}\}}{{\rm{\Phi }}}_{{\rm{a}}}({\omega }_{1},{\omega }_{2};{{\bf{q}}}_{1},{{\bf{q}}}_{2}){{\rm{\Phi }}}_{{{\rm{a}}}^{\prime}}^{\ast }({\omega }_{1}^{\prime},{\omega }_{2}^{\prime};{{\bf{q}}}_{1}^{\prime},{{\bf{q}}}_{2}^{\prime})\\  &  & \times |{{\rm{a}}}_{1},{{\rm{a}}}_{2}\rangle\langle{{\rm{a}}}_{1}^{\prime},{{\rm{a}}}_{2}^{\prime}|\langle{\omega }_{1}^{\prime};{{\bf{q}}}_{1}^{\prime}|\langle{\omega }_{2}^{\prime};{{\bf{q}}}_{2}^{\prime}|,\end{array}$$

where the superscript ^*^ denotes the complex conjugate. Tracing out the frequency and momentum, the reduced density matrix of the produced polarization two-photon state is

25 $$\begin{array}{ccc}{\rho }^{\sigma } & = & {{\rm{T}}{\rm{r}}}_{{\bf{q}}}[{{\rm{T}}{\rm{r}}}_{\omega }(\rho )]\\  & = & \int d{{\bf{q}}}_{1}^{\prime\prime}d{{\bf{q}}}_{2}^{\prime\prime}\langle{{\bf{q}}}_{1}^{\prime\prime},{{\bf{q}}}_{2}^{\prime\prime}|\int d{\omega }_{1}^{\prime\prime}d{\omega }_{2}^{\prime\prime}\langle{\omega }_{1}^{\prime\prime},{\omega }_{2}^{\prime\prime}|\rho |{\omega }_{1}^{\prime\prime},{\omega }_{2}^{\prime\prime}\rangle|{{\bf{q}}}_{1}^{\prime\prime},{{\bf{q}}}_{2}^{\prime\prime}\rangle\\  & = & \int d{\omega }_{1}d{\omega }_{2}d{{\bf{q}}}_{1}d{{\bf{q}}}_{2}{\sum }_{{\rm{a}},{{\rm{a}}}^{\prime}=\{{\rm{H}},{\rm{V}}\}}{{\rm{\Phi }}}_{{\rm{a}}}({\omega }_{1},{\omega }_{2};{{\bf{q}}}_{1},{{\bf{q}}}_{2}){{\rm{\Phi }}}_{{{\rm{a}}}^{\prime}}^{\ast}({\omega }_{1},{\omega }_{2};{{\bf{q}}}_{1},{{\bf{q}}}_{2})|{{\rm{a}}}_{1},{{\rm{a}}}_{2}\rangle\langle{{\rm{a}}}_{1}^{\prime},{{\rm{a}}}_{2}^{\prime}|\end{array}$$

where *σ*, **q**, and *ω* refer to the polarization, momentum, and frequency degrees of freedom, respectively. The nonzero elements of the reduced state are thus ${\rho }_{{\rm{HH}},{\rm{HH}}}^{\sigma }={\rho }_{{\rm{VV}},{\rm{VV}}}^{\sigma }=\frac{1}{2}$ and the off-diagonal elements ${\rho }_{{\rm{H}}{\rm{H}},{\rm{V}}{\rm{V}}}^{\sigma }={({\rho }_{{\rm{V}}{\rm{V}},{\rm{H}}{\rm{H}}}^{\sigma })}^{\ast }$ are as expressed in equation (10).

If the frequency and polarization are traced out from the pure state (1), the resulting spatial two-photon state is described by the reduced density matrix

26 $$\begin{array}{rcl}{\rho }^{{\bf{q}}} & = & {{\rm{Tr}}}_{\sigma }[{{\rm{Tr}}}_{\omega }(\rho )]\\  & = & {\sum }_{{{\rm{a}}}_{1}^{\prime\prime},{\rm{a}}_{2}^{\prime\prime}=\{H,V\}}\langle {{\rm{a}}}_{1}^{\prime\prime},{{\rm{a}}}_{2}^{\prime\prime}|\int d{\omega }_{1}^{\prime\prime}d{\omega }_{2}^{\prime\prime}\langle {\omega }_{1}^{\prime\prime},{\omega }_{2}^{\prime\prime}|\rho |{\omega }_{1}^{\prime\prime},{\omega }_{2}^{\prime\prime}\rangle |{{\rm{a}}}_{1}^{\prime\prime},{{\rm{a}}}_{2}^{\prime\prime}\rangle \\  & = & \int d{\omega }_{1}d{\omega }_{2}d{{\bf{q}}}_{1}d{{\bf{q}}}_{2}d{{\bf{q}}}_{1}^{\prime}d{{\bf{q}}}_{2}^{\prime}\\  &  & \times [{{\rm{\Phi }}}_{{\rm{H}}}({\omega }_{1},{\omega }_{2};{{\bf{q}}}_{1},{{\bf{q}}}_{2}){{\rm{\Phi }}}_{{\rm{H}}}^{\ast }({\omega }_{1},{\omega }_{2};{{\bf{q}}}_{1}^{\prime},{{\bf{q}}}_{2}^{\prime})\\  &  & +{{\rm{\Phi }}}_{{\rm{V}}}({\omega }_{1},{\omega }_{2};{{\bf{q}}}_{1},{{\bf{q}}}_{2}){{\rm{\Phi }}}_{{\rm{V}}}^{\ast }({\omega }_{1},{\omega }_{2};{{\bf{q}}}_{1}^{\prime},{{\bf{q}}}_{2}^{\prime})]|{{\bf{q}}}_{1},{{\bf{q}}}_{2}\rangle \langle {{\bf{q}}}_{1}^{\prime},{{\bf{q}}}_{2}^{\prime}|\end{array}$$

The corresponding state purity after frequency filters is then as given by equation (14).

### HOM-type interferometer for orthogonal amplitudes of nondegenerate, noncollinear SPDC

The state at the input ports *A*, *B* of the beam splitter is expressed as

27 $$\begin{array}{c}\frac{1}{2}\int d{\omega }_{1}d{\omega }_{2}[{{\rm{e}}}^{-i{\omega }_{1}\tau }{{\rm{\Phi }}}_{{\rm{H}}}^{{\rm{o}}}({\omega }_{1},{\omega }_{2}){\hat{a}}_{A,H}^{+}({\omega }_{1}){\hat{a}}_{B,H}^{+}({\omega }_{2})+{{\rm{e}}}^{-i{\omega }_{2}\tau }{{\rm{\Phi }}}_{{\rm{V}}}^{{\rm{o}}}({\omega }_{2},{\omega }_{1}){\hat{a}}_{A,H}^{+}({\omega }_{2}){\hat{a}}_{B,H}^{+}({\omega }_{1})\\ +{{\rm{e}}}^{-i{\omega }_{1}\tau }{{\rm{\Phi }}}_{{\rm{V}}}^{{\rm{o}}}({\omega }_{1},{\omega }_{2}){\hat{a}}_{A,V}^{+}({\omega }_{1}){\hat{a}}_{B,V}^{+}({\omega }_{2})+{{\rm{e}}}^{-i{\omega }_{2}\tau }{{\rm{\Phi }}}_{{\rm{H}}}^{{\rm{o}}}({\omega }_{2},{\omega }_{1}){\hat{a}}_{A,V}^{+}({\omega }_{2}){\hat{a}}_{B,V}^{+}({\omega }_{1})]|\rangle,\end{array}$$

where ${\hat{a}}_{i,j}^{+}({\omega }_{k})$ is the creation operator in the spatial mode *i*, for polarization *j*, and at angular frequency *ω*_*k*_, $|0\rangle$ is the vacuum state. We assume a symmetric beam splitter. Substituting ${\hat{a}}_{A}^{+}=({\hat{a}}_{C}^{+}+i{\hat{a}}_{D}^{+})/\sqrt{2}$ and ${\hat{a}}_{B}^{+}=(i{\hat{a}}_{C}^{+}+{\hat{a}}_{D}^{+})/\sqrt{2}$ (*C* and *D* label the output ports of the beam splitter) and using the commutation relation: $[{\hat{a}}^{+}({\omega }_{k}),{\hat{a}}^{+}({\omega }_{k^{\prime} })]=\delta ({\omega }_{k}-{\omega }_{k^{\prime} })$, the exiting state after the beam splitter writes

28 $$\begin{array}{c}|{{\rm{\Psi }}}_{{\rm{o}}{\rm{u}}{\rm{t}}}\rangle=\frac{1}{4}\int d{\omega }_{1}d{\omega }_{2}\{[{{\rm{e}}}^{-i{\omega }_{1}\tau }{{\rm{\Phi }}}_{{\rm{H}}}^{{\rm{o}}}({\omega }_{1},{\omega }_{2})+{{\rm{e}}}^{-i{\omega }_{2}\tau }{{\rm{\Phi }}}_{{\rm{V}}}^{{\rm{o}}}({\omega }_{2},{\omega }_{1})][i{\hat{a}}_{C,H}^{+}({\omega }_{1}){\hat{a}}_{C,H}^{+}({\omega }_{2})+\,i{\hat{a}}_{D,H}^{+}({\omega }_{1}){\hat{a}}_{D,H}^{+}({\omega }_{2})]\\ \, +[{{\rm{e}}}^{-i{\omega }_{1}\tau }{{\rm{\Phi }}}_{{\rm{H}}}^{{\rm{o}}}({\omega }_{1},{\omega }_{2})-{{\rm{e}}}^{-i{\omega }_{2}\tau }{{\rm{\Phi }}}_{{\rm{V}}}^{{\rm{o}}}({\omega }_{2},{\omega }_{1})][{\hat{a}}_{C,H}^{+}({\omega }_{1}){\hat{a}}_{D,H}^{+}({\omega }_{2})-{\hat{a}}_{C,H}^{+}({\omega }_{2}){\hat{a}}_{D,H}^{+}({\omega }_{1})]\\ \, +[{{\rm{e}}}^{-i{\omega }_{1}\tau }{{\rm{\Phi }}}_{{\rm{V}}}^{{\rm{o}}}({\omega }_{1},{\omega }_{2})+\,{{\rm{e}}}^{-i{\omega }_{2}\tau }{{\rm{\Phi }}}_{{\rm{H}}}^{{\rm{o}}}({\omega }_{2},{\omega }_{1})][i{\hat{a}}_{C,V}^{+}({\omega }_{1}){\hat{a}}_{C,V}^{+}({\omega }_{2})+i{\hat{a}}_{D,V}^{+}({\omega }_{1}){\hat{a}}_{D,V}^{+}({\omega }_{2})]\\ \,+[{{\rm{e}}}^{-i{\omega }_{1}\tau }{{\rm{\Phi }}}_{{\rm{V}}}^{{\rm{o}}}({\omega }_{1},{\omega }_{2})-{{\rm{e}}}^{-i{\omega }_{2}\tau }{{\rm{\Phi }}}_{{\rm{H}}}^{{\rm{o}}}({\omega }_{2},{\omega }_{1})][{\hat{a}}_{C,V}^{+}({\omega }_{1}){\hat{a}}_{D,V}^{+}({\omega }_{2})-{\hat{a}}_{C,V}^{+}({\omega }_{2}){\hat{a}}_{D,V}^{+}({\omega }_{1})]\}|\rangle,\end{array}$$

which leads directly to the coincidence probability in equation (17).
